# Decoding (digital) histopathology: The building blocks for computational researchers

**DOI:** 10.1371/journal.pdig.0001148

**Published:** 2026-05-13

**Authors:** Salma Dammak, Alessandro Caputo, Diana Montezuma, Vincenzo L’Imperio, Sara P. Oliveira, on behalf of the European Society of Digital and Integrative Pathology (ESDIP)

**Affiliations:** 1 European Society of Digital and Integrative Pathology (ESDIP), Lisbon, Portugal; 2 Computational Pathology Group, Radboud University Medical Center, Nijmegen, The Netherlands; 3 Department of Medicine, Surgery and Dentistry ‘Scuola Medica Salernitana’, University of Salerno, Salerno, Italy; 4 Department of Pathology, University Hospital of Salerno, Salerno, Italy; 5 Research & Development Unit, IMP Diagnostics, Porto, Portugal; 6 Cancer Biology and Epigenetics Group, Research Center of IPO Porto (CI-IPOP), Portuguese Oncology Institute of Porto (IPO Porto), Porto Comprehensive Cancer Center Raquel Seruca (Porto.CCC) & CI-IPOP@RISE (Health Research Network), Porto, Portugal; 7 School of Medicine and Surgery, University of Milano-Bicocca, Monza, Italy; 8 Department of Pathology, Fondazione IRCCS San Gerardo dei Tintori, Monza, Italy; 9 Computational Pathology Group, Department of Pathology, The Netherlands Cancer Institute, Amsterdam, The Netherlands; University of Washington, UNITED STATES OF AMERICA

## Abstract

Computational Pathology is a novel discipline at the intersection of pathology and computer science, driven by the recent advances in machine learning and image analysis. Nevertheless, combining the insights from both disciplines remains challenging, particularly due to differences in technical background and language between pathologists and engineers. It is acknowledged that literature translating fundamental pathology concepts for computer scientists remains limited, which further complicates the understanding of the field, especially for those entering the field. In this context, and aligned with the mission of the European Society of Digital and Integrative Pathology (ESDIP) to promote education and interdisciplinary collaboration in digital and computational pathology, this work aims to provide a comprehensive yet accessible guide to pathology for computational scientists and other researchers. Herein, we present an overview of the pathology laboratory workflow, digital pathology and whole-slide imaging, diagnostic fundamentals of neoplastic and nonneoplastic diseases, and current applications of AI in pathology. This guide is designed as a practical reference and educational resource to support computer scientists new to the field and to promote more effective collaboration between medical and computational communities.

## Author summary

Computational pathology brings together pathology and artificial intelligence to analyse digitised tissue slides, yet researchers entering the field often struggle with its medical terminology, laboratory processes, and diagnostic logic. This review provides an accessible guide to the essential building blocks of pathology for computational scientists. We describe how tissue moves from the patient to a glass slide, highlighting how fixation, embedding, cutting, and staining shape the final image and introduce artefacts that influence algorithm performance. We then explain how glass slides become whole slide images, outlining key technical concepts such as pyramidal image structures, magnification versus resolution, scanner architectures, and data storage constraints. Beyond image acquisition, we introduce the fundamentals of diagnostic reasoning, including the importance of clinical context, low-to-high magnification examination strategies, and common interpretative pitfalls. Finally, we map these principles to core computational tasks - segmentation, diagnosis and prognosis modelling, and discovery research - together with supportive workflows and practical challenges. By establishing a shared conceptual framework, this guide aims to facilitate interdisciplinary collaboration and support the responsible development of artificial intelligence tools in pathology.

## Introduction

Pathology, from the Greek *pathos* and *logia*, literally refers to the study of suffering or disease [1,2]. Since the 19th century, when autopsy specimens were coveted and clandestine autopsies were common, pathology has evolved into a modern discipline, with insights that extend beyond the traditional diagnostic purposes and are crucial for treatment prediction and patient prognosis. While “Pathology” encompasses various sub-disciplines, this guide focuses specifically on **histopathology**—the study of solid tissue architecture. While other areas like cytopathology (the study of individual cells) are equally vital and increasingly digitized, they involve radically different laboratory workflows and computational challenges that fall outside the scope of this manuscript. More recently, Computational Pathology (CPath) has emerged as a novel discipline at the intersection of pathology and computer science. Traditionally separated fields, these disciplines now converge to integrate artificial intelligence (AI)-based image analysis into the examination of histological tissue samples. One difficulty lies in the challenge these professionals face in fully comprehending each other’s language and methodologies. This poses a particular challenge for those newly entering the field, who must navigate complex scientific and technical vocabularies and may lack interdisciplinary insights. Specifically, it was recently noted by Mandal et al. [3] that literature translating basic pathology concepts for computer scientists and AI developers remains scarce. The authors have published a tumour histopathology glossary aimed at expanding the computational community’s knowledge in this domain [3]. In line with this study and recognising the persistent gap in the field, our work focuses on further contributing to improving access to fundamental pathology topics for the computational community. Herein, we aim to provide a comprehensive, yet accessible, guide for computer scientists and other researchers seeking to understand the essentials of histopathology. Coming from a multidisciplinary team that has first-hand experience with these interdisciplinary challenges, this guide is written in a clear and concise way to serve as a practical reference or syllabus for professionals new to pathology, promoting effective multidisciplinary collaboration.

Besides this general introduction, this guide is structured into four main sections, designed to accompany the reader from tissue handling to computational modelling:

- **Section 2** provides an overview of the histopathology laboratory workflow, following the journey of a specimen from tissue collection to the production of a stained glass slide, and highlighting how pre-analytical and analytical steps shape the final image and introduce artefacts;
- **Section 3** introduces Digital Pathology and whole slide imaging, explaining the technical foundations of slide digitisation, image structure, magnification and resolution, scanner architectures, and data storage considerations;
- **Section 4** summarises the fundamentals of diagnostic reasoning, outlining how pathologists interpret slides across magnification scales, integrate clinical and macroscopic context, and navigate common pitfalls in neoplastic and non-neoplastic disease;
- **Section 5** discusses CPath, mapping diagnostic tasks to AI methodologies, including segmentation, diagnosis, and prognosis modelling, discovery tasks, supportive workflows, and key challenges in model development and deployment.

## Pathology lab essentials: from tissue to glass slides

**Fig 1:**
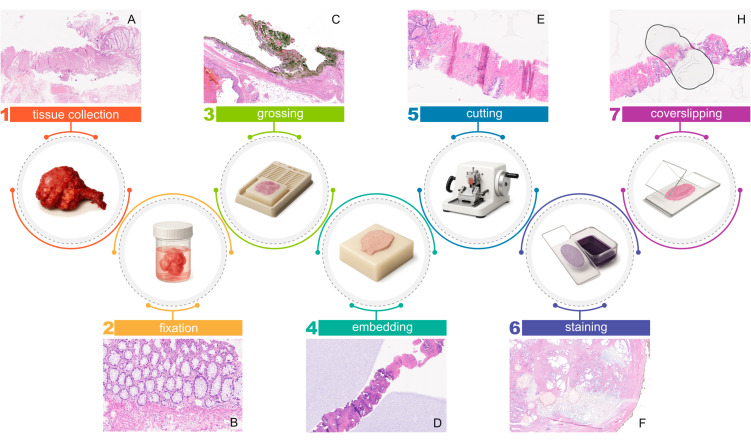
The main steps of the pathology workflow (circles) coupled with an image showing an artefact derived from each step (rectangles). From left to right: 1 – tissue collection, (A) cautery effect; 2 – fixation, (B) underfixation; 3 – grossing, (C) ink; 4 – embedding, (D) gel matrix; 5 – cutting, (E) tissue folds; 6 – staining, (F) uneven staining; 7 – coverslipping, (H) air bubble. The images in this figure were generated using the AI-based tool Illustrae (https://illustrae.co). The content was reviewed and curated by the authors to ensure scientific accuracy and appropriate representation.

The transformation of biological tissue into a digital image is a multi-step process (Fig 1) involving various laboratory professionals. Generally, the physical handling of the specimen—including fixation, processing, embedding, and staining—is performed by **biomedical laboratory scientists and technicians**. The **pathologist** typically performs the macroscopic examination (grossing) and provides the final microscopic interpretation. For the computational researcher, understanding that the final image is the result of standardized but manual laboratory craftsmanship is essential for contextualizing the technical variability discussed below; decisions made during tissue handling at every stage irreversibly shape what is eventually visualised, potentially introducing artefacts (Table A in Appendix S1) with which computational models must contend.

### Tissue collection

Tissues submitted to pathology are typically classified by the collection procedure. Biopsies are generally incisional, meaning they involve removing only a small portion of tissue from a lesion to aid in diagnosis, and usually do not fully remove the abnormal area (radicality). Therefore, they are not curative, and final treatment decisions usually depend on the biopsy results. In contrast, excisional biopsies and surgical specimens (also called resections) involve removing the entire lesion or area of interest, providing enough tissue for definitive diagnosis and aiming for curative radicality with clear margins (the edge of healthy tissue around the removed lesion).

Side effects of both biopsy and surgical procedures can induce artefacts within the samples, such as **cautery and crush artefacts** (Fig 1A, and Table A and Figure A in Appendix S1).

The collected fresh samples are unsuitable for microscopic analysis, and once removed from the organism’s blood supply, they quickly start to degenerate. Hence, tissue samples must undergo a series of processes to stop degeneration and prepare them for microscopic examination.

### Fixation

**Fixation** halts autolysis and degradation, stabilising both the tissue’s morphological architecture and its molecular components (*e.g.*, proteins and nucleic acids) for downstream analyses. Furthermore, fixed tissue acquires a harder consistency and is thus easier to cut in further steps. Different fixatives can be used, but the universally recognised medium for tissue fixation is an aqueous solution of formaldehyde, which preserves the tissues and cells through the creation of cross-links among proteins [4]. **Underfixation** [5] and **overfixation** complicate tissue preparation, potentially affecting downstream analysis (*e.g.,* immunohistochemistry (IHC) for predictive markers [6,7]). Additionally, underfixation corrupts tissue and cell morphology (Fig 1B).

### Grossing

During the **grossing** step, the tissue samples are observed, described, and any alterations are noted and measured—**macroscopic examination**—and then selected (how many and which parts) to process for microscopic analysis—**sampling**—depending on the type of specimen and macroscopic findings. **Inking** can be used to mark certain parts of the tissue, commonly surgical margins, to make it recognisable on the final slide (Fig 1C). It is important to understand that for large specimens (*e.g.*, an entire lung or colon), it is diagnostically not useful and economically infeasible to sample the entire organ. Therefore, sampling is strategic and guided by clinical and radiological findings to ensure the most relevant areas are captured.

### Tissue processing and embedding

**Tissue processing** consists of tissue dehydration, through a series of immersions in alcohol and xylene, followed by paraffin wax infiltration. The tissue sample is then encased in a block of paraffin wax - **tissue embedding** - to stiffen and give it a homogeneous consistency, essential for microtome cutting.

It is important to note that during this stage, the spatial orientation of the tissue is fixed. Any auxiliary materials used during processing to secure the sample, such as biopsy sponges or papers, may be left around the tissue and be visible in the final WSI (Fig 1D).

*Actionable insight:* The orientation fixed during embedding determines the 2D plane of the final digital image; if a specific structure is not oriented correctly in the block, it may be visible suboptimally (or not at all) in the resulting WSI.

### Microtome cutting

Once embedded in paraffin blocks, the tissue is sectioned using a microtome. Sections are typically cut at a thickness of 2–5 microns (µm), parallel to the block surface; sections that are too thick cause overlapping nuclei and loss of detail, while those too thin may lose structural integrity. These thin slices are then floated on a water bath and mounted onto glass slides.

Artefacts introduced during this stage can manifest as structural noise in the obtained digital slides after scanning (*e.g.*, tissue folds, as in Fig 1E), complicating image analysis algorithms. Moreover, some of these can negatively impact the scanning phase by causing poor-quality (out-of-focus) images, ultimately leading to altered interpretation by AI algorithms [8].

*Actionable insight:* AI developers should treat these structural artifacts not merely as noise to be filtered, but as a source of data variability. Implementing heavy augmentation or developing automated quality control filters to exclude out-of-focus tiles is essential to ensure model reliability in clinical settings [8].

### Staining

**Fig 2:**
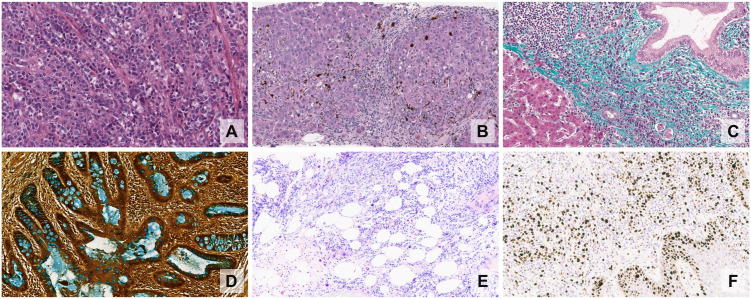
Examples of common stains: (A) H&E, (B) PAS, (C) Masson trichrome, (D) Alcian blue, (E) Giemsa, (F) Ki67 IHC. Examples from the University of Leeds Virtual Pathology repository [9].

The unstained slices are mostly transparent, except for some intrinsic pigments. Consequently, **staining** is required to extract any significant morphological information and highlight different physical, chemical, or biomolecular characteristics of tissue. The most common routine staining technique using **haematoxylin and eosin (H&E)**, which highlights nuclei (and other basophilic structures) in blue-purple, and cytoplasm and extracellular matrix proteins pink, respectively (Fig 2).

In addition to H&E (Fig 2A), several other stains are used, with the most common being the plethora of histochemical and immunohistochemical stains. **Histochemical stains**, such as periodic acid-Schiff (PAS), Masson trichrome, Alcian blue or Giemsa (Fig 2B–E, respectively), exploit some physical and chemical properties of tissues to stain them in a meaningful way, just like H&E. **Immunohistochemical stains**, on the other hand, leverage the very specific antibody-antigen interaction to highlight the presence of certain proteins. These interactions are visualised using chromogenic substrates (*e.g.*, diaminobenzidine - DAB), producing a visible precipitate (brown, in the case of DAB), such as the example in Fig 2F.

Staining artefacts are mostly related to the variability of the chromatic properties of a stain (Fig 1F) across laboratories or even within the same laboratory. While modern laboratories often use **fully automated staining platforms**, these improve consistency but do not fully eliminate variability, since innumerable variables affect the final color, leading to both regional and temporal differences.

*Actionable insight:* Because staining variability is both regional and operator-dependent, developers must not rely solely on digital stain normalization. Models should be validated, and ideally trained, on multi-center data with diverse staining profiles rather than assuming a “standard” H&E appearance.

### Coverslipping

**Coverslipping** serves the dual purpose of protecting the tissue on the slide from damage and of homogenising the light refractive properties of the tissue within the glass slide. Conventionally, the tissue placed on the glass slide is covered by a second, thinner piece of glass. A mounting medium, which polymerises and hardens, much like a glue, is put between the two pieces of glass and around the tissue. Some alternative methods, however, use a plastic film, a layer of polymer, or other materials as the coverslip. In high-throughput laboratories, this process is typically automated. However, artefacts can still emerge due to the presence of variability in mounting media, such as bubbles (Fig 1H) and regions of the tissue beyond the coverslip (thus not mounted), appearing as dark, opaque, and blurred regions in the digital image due to the refractive index mismatch.

*Human vs. machine perception:* It is important to note that while the laboratory steps described above can introduce artifacts, they rarely pose an insurmountable diagnostic limitation for a pathologist. In routine practice, if an artifact jeopardizes an assessment, in most (but not all) cases, the slide can be amended (*e.g.,* re-coverslipped) or re-done (*e.g.*, by recutting a new section from the paraffin block).

*Actionable insight:* For the AI developer, however, these artifacts represent significant computational limitations. Unlike a pathologist, an algorithm typically operates on the digital data provided without the ability to request a better sample. Therefore, what is a minor procedural inconvenience in a clinical workflow becomes a hard failure point for an AI model. Developers should frame artifact-heavy slides as a challenge of algorithmic robustness rather than a lack of diagnostic information in the underlying tissue.

### Beyond histopathology

As the field matures, the principles outlined here for tissue sections will need to be adapted for cytopathology. AI developers should be aware that cytology samples - such as fine-needle aspirates or smears - present distinct challenges, including sparse cellularity, overlapping cells in different focal planes, and varying background artifacts. These topics represent a significant portion of the pathology workload and warrant a dedicated computational syllabus in the future.

## Digital pathology: from glass slides to images

Once the glass slides are prepared, they can be assessed under the microscope, in the traditional pathology workflow, or they can be digitised into high-resolution digital images, **whole slide images (WSIs)**, using slide scanners. The digital slides can then be viewed, analysed, and stored using specialised software. In modern pathology, the shift from analogue to digital is more than technological. It is driven by the need for more efficient and accurate diagnostic workflows and represents a paradigm change in how diagnostic medicine is delivered [10,11].

Traditional glass slides are prone to physical degradation, loss, and limited accessibility. With digitisation, laboratories can improve data preservation, facilitate collaboration among pathologists (worldwide), and enable the development and implementation of AI-powered diagnostic tools to enhance accuracy and speed [12,13]. Additionally, **digital pathology** allows for streamlined storage, easy retrieval of patient data, and integration with other modalities (such as radiology and molecular studies) [12].

In the clinical setting, digitisation supports faster second opinions and multidisciplinary collaboration. In research, it allows easy annotation, labelling, and sharing of datasets essential for AI model training [14].

### Whole slide images

Whole slide imaging [15] is a cornerstone of digital pathology, enabling the visualisation of entire histological slides on a screen at high resolution. As the digital counterpart of a microscope, it is important to understand some of its technical specificities, particularly when designing digital pathology workflows or developing and deploying AI tools.

#### Pyramidal image structure.

**Fig 3:**
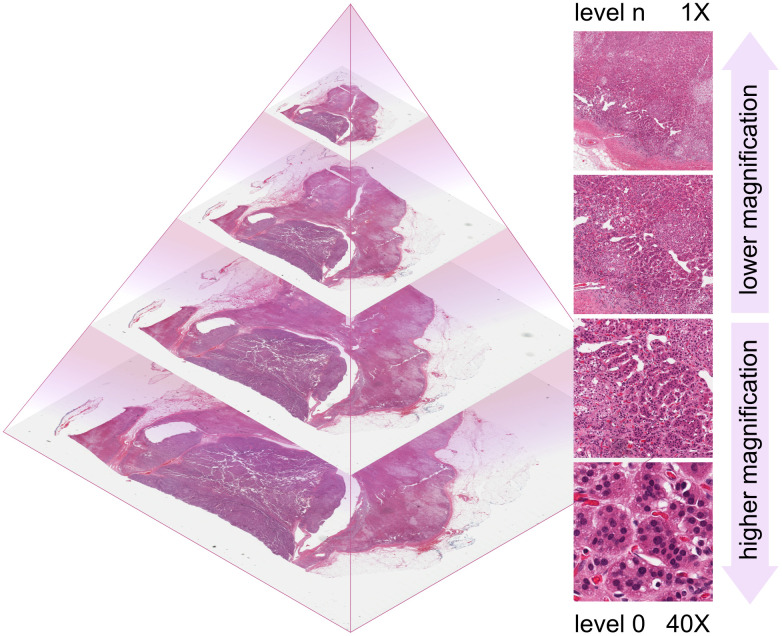
Whole slide image (WSI) pyramidal structure. Lower levels correspond to higher magnifications, thus higher resolutions, and vice versa. Example from The Cancer Genome Atlas (TCGA) dataset [16].

WSI files are typically stored in a pyramidal image structure (Fig 3), in which multiple resolution levels are embedded within a single file. This design allows smooth zooming in and out (much like navigating digital maps) without needing to reload or decompress the entire image, since each level is downsampled from the base layer. Such a structure mimics the traditional experience of examining slides under a microscope, where a pathologist adjusts magnification dynamically to focus on areas of interest. Moreover, it also improves memory efficiency and processing speed, critical for both human experience and AI inference.

#### Optical magnification vs. pixel pitch.

Optical magnification (*e.g.*, 20× or 40×) refers to the physical level by which an image is optically enlarged by the objective system. However, AI researchers must distinguish between optical resolution and digital sampling. **Optical resolution** is a physical limit determined by the objective lens’s Numerical Aperture (NA); it defines the minimum distance at which two distinct points can be distinguished. In contrast, **pixel pitch** (or sampling period, often expressed as microns per pixel, µm/pixel) defines how much tissue detail is captured per digital pixel. While scanners may share the same optical magnification, the final pixel pitch can differ due to variations in sensor quality, size, and acquisition technology. For example, two scanners may operate at 40×, but one produces images at 0.5 µm/pixel while the other achieves 0.25 µm/pixel. It is worth noting that if an image is blurry because it is out of focus due to optical issues (*e.g.*, suboptimal z-level), neither increasing the optical magnification nor the pixel pitch will improve the image.

*Actionable insight:* While pixel pitch can be arbitrarily changed through upsampling or downsampling, the underlying optical resolution remains fixed by the hardware. AI developers should be cautious: a 0.25 µm/pixel image from a low-NA scanner will be blurrier than one from a high-NA scanner, despite having the same digital dimensions. Higher magnification and finer pixel pitch increase diagnostic detail but significantly increase data volume. Understanding these trade-offs is essential, as different pathology tasks (*e.g.*, tumour classification vs. mitosis detection) require different image scales and resolutions for optimal performance. When documenting datasets, both the magnification/NA of the objective and the pixel pitch (µm/pixel) should be reported to ensure model reproducibility.

#### Scanner architecture.

Tile (or area) scanners acquire images in discrete sections that are later stitched together, enabling faster throughput. However, this process can introduce **stitching artifacts**, such as visible “seams” or misalignments where tiles meet. In contrast, line scanners capture slides line-by-line, offering high fidelity and fewer stitching artifacts, though they may still be susceptible to longitudinal banding.

*Actionable insight:* Stitching artifacts can significantly impact AI performance, particularly for segmentation tasks. A model might incorrectly detect a “line” or edge at a tile boundary, or fail to recognize a single cell that has been physically bisected or misaligned by the stitching algorithm. Developers should ensure model performance is robust to tile edges to mitigate the risk of missing features located on these artificial boundaries.

#### Data formats, compression, and storage.

While some slide formats are open and standardised, such as TIFF or DICOM, many are proprietary, such as SVS, MRXS, or NDPI [14]. The sheer size of WSI data, especially when compared to data from other imaging modalities, such as radiology [17], makes data compression a critical aspect for efficient storage. With a single slide (from a large sample, scanned at 40×) likely to have more than 100,000 × 100,000 pixels and exceed 10 GB, compression methods like JPEG, JPEG2000, or LZW help reduce storage demands, but must preserve diagnostic integrity [18]. Scalable and high-performance storage solutions (often integrated with cloud infrastructure) are essential to support long-term and cost-effective archiving, rapid retrieval, and AI processing at scale.

### Advantages & challenges of slide digitisation

**Fig 4:**
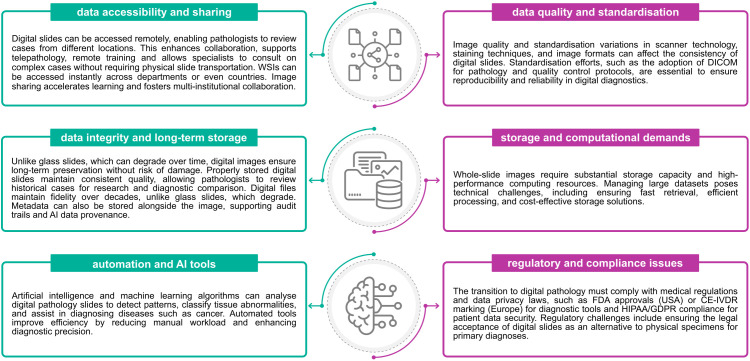
Advantages and challenges of digital transition in Pathology.

The usage of WSI represents a transformative step in modern diagnostic workflows, offering numerous advantages over traditional glass slides. It improves accessibility, collaboration, and long-term data preservation through remote access, AI-assisted analysis, and secure metadata storage. However, the digital transition introduces some challenges, such as image quality and standardisation, high storage and computing demands, and strict regulatory and compliance requirements for diagnostic use (Fig 4). Balancing both sides is essential, not only for the successful and scalable implementation of digital workflows in clinical practice but also for the development of efficient and useful AI tools.

## Fundamentals of diagnosis: from images to diagnostic patterns

Based on glass slides and/or their corresponding WSI, (digital) pathology is fundamentally a visually driven discipline, with pathologists extracting and recognising diagnostic patterns from the complex images of tissue samples. However, diagnostic reasoning extends beyond the slide, integrating clinical context, macroscopic and microscopic information, and molecular assays towards a complete diagnostic picture and pathology report.

### Basic diagnostic orientation

Before examining the slide, pathologists ground their interpretation in contextual information. The **clinical context** is fundamental to anchor the diagnostic hypothesis. Especially in **nonneoplastic disease**, *i.e.,* inflammatory/autoimmune/infective or maladaptive disorders of organs and tissues, the same slide can be diagnostic for two different conditions, depending on clinical context (*e.g.,* ulcerative colitis vs. segmental colitis associated with diverticula [SCAD]). Even in **neoplastic disease**, *i.e.,* abnormal tissue growth resulting from uncontrolled cell growth, patient history (*e.g.,* smoking or prior malignancy) drastically reshapes the expected spectrum of disease. Then the first step is to correlate what is seen in the slide with the **macroscopic description** and the material within the paraffin block, ensuring that the slide corresponds to the intended tissue (*e.g.,* tumour, surgical margin, lymph node). Especially in fragmented specimens like biopsies, this confirmation step is essential to avoid misinterpretation. Tracking systems help document and verify specimen-slide correspondence, aiding both quality control and data integrity, which is also essential for AI development [19].

*Actionable insight:* Developers must recognize where AI algorithms **can not** currently be fitted into a workflow:

- **Primary diagnosis without clinical context:** AI should not be tasked with providing a definitive diagnosis for nonneoplastic diseases where the histology is identical, but the clinical history (*e.g.*, drug use vs. infection) is the deciding factor.
- **Incomplete sampling assessment:** An AI model cannot confirm “clear margins” if the surgeon or pathologist did not sample the actual surgical edge during grossing.
- **Fragmented specimens:** In fragmented biopsies, AI models may struggle with orientation; developers should avoid projects requiring precise spatial localization unless some sort of orientation is provided to the model.

Understanding these “can not” scenarios prevents the development of “black-box” models that might make high-confidence predictions based on incomplete biological data.

### Slide examination strategy: from low to high power

**Fig 5:**
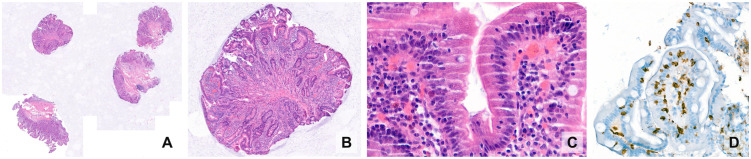
The pathologists’ examination strategy: different information is gathered at different magnifications. Tissue-level aspects such as architecture are best assessed at low and very low power **(A, B)**, whereas cell-level details require high power **(C, D)**. Haematoxylin&Eosin (A–C) and diaminobenzidine immunohistochemistry for CD3 (D).

The slide examination proceeds from the overview, at the **architectural level**, in a low-power magnification, to the detail at the **cellular level**, in a high-power magnification (Fig 5). Using the 2× or 4× magnification, pathologists evaluate overall architecture. In tumours, they assess lesion boundaries (infiltrative/irregular vs pushing/well-defined), necrosis, cellularity gradients, and hotspot regions (*e.g.,* for mitotic count). In nonneoplastic specimens, general patterns (fibrosis, inflammation, atrophy) are noted, and adequacy is assessed (*e.g.,* number of glomeruli in a kidney biopsy or portal tracts in a liver core). Higher magnifications of 20× or 40× are used to interrogate specific structures, such as nuclear atypia, mitoses, inflammatory cell types, and inclusion bodies, that become evident (further described in the tumour glossary by Mandal S. et al. [3]). Immunohistochemistry or special stains may follow to assess specific biomarkers, but this level provides most morphologic discriminants.

### Common pitfalls and cognitive biases

The digital advent is transforming pathologists’ practice by enabling greater scalability, collaboration, and ultimately, computational analysis. However, despite such advancements, pathology assessment is susceptible to errors influenced by perception, experience, and cognitive bias. The digital medium does not eliminate these vulnerabilities, and in some cases, it may even exacerbate them. Thus, when developing reliable AI tools, it is crucial to recognise and understand the common pitfalls to maintain diagnostic accuracy and, ultimately, ensure patient safety. In a field where minor oversights can have significant consequences (*e.g.,* missing a high-grade tumour or deciding on the wrong treatment), critical awareness is as important as technical innovation.

#### Mislabelling or mislocalization of features.

A single slide rarely presents a uniform pathology; it often contains a complex mixture of tissue heterogeneity, including normal tissue, premalignant lesions, and invasive malignancy simultaneously. Furthermore, it is critical to recognize that a definitive diagnosis for a patient may not come from the specific slide being viewed, but from a different tissue block entirely.

Another related potential pitfall is scoring biomarkers or dysplasia on non-target cells. For example, a high Ki-67 index is normally found in reactive (regenerating) benign cells and should not be misinterpreted as dysplasia or neoplasia [20]. Some important biomarkers that guide therapy (*e.g.,* HER2 overexpression in breast cancer [21]) are often overexpressed in precursor lesions (ductal carcinoma *in situ*), and this is of no diagnostic relevance since the biomarker should be evaluated in true cancerous cells [22].

*Actionable insight:* High-level slide labels (*e.g.*, “cancer”) should be handled using multiple instance learning (MIL) or similar weakly supervised frameworks rather than assigning the slide label to every constituent patch, which introduces significant label noise. When curating datasets, researchers must ensure that the “ground truth” labels are derived from the specific slide or block being used, rather than a generic patient-level diagnosis that may not be reflected in the sampled tissue.

#### Overlooking clinical or gross context.

Disregarding clinical history or failing to correlate with the gross description can lead to misdiagnosis, for example, mistaking tumour recurrence for a new primary in the absence of clinical data.

#### Diagnostic summary and report generation.

The final diagnosis is not simply a microscopic description, but a clinical integration.

#### Synthesis of visual and contextual data.

Pathologists weigh morphologic patterns with clinical context, gross features, and prior history. Assistive AI systems can work well without such context, such as those generating heatmaps for features of interest (*e.g.,* mitoses or tumour areas) to guide the pathologist. On the other hand, end-to-end diagnostic AI systems must eventually emulate this synthesis step.

#### Communication of diagnostic certainty and next steps.

Reports often include levels of certainty (*e.g.,* “suggestive of,” “consistent with”), differential diagnoses, and suggestions for further testing. This communicates the diagnostic logic and acknowledges uncertainty.

## Computational Pathology: from diagnostic patterns to AI models

In pathology, AI addresses two main challenges: managing high workload demands with the concurrent global shortage of trained pathologists [23] and improving diagnostic quality despite inherent uncertainties. To reduce workload, AI models automate time-consuming tasks such as slide review and tissue segmentation. To improve quality, they aim to match or exceed the performance of pathology experts, reduce inter-observer variability, and enhance accuracy in rare or complex cases, especially when certain diagnostic data (*e.g.*, special stains, molecular or genetic testing) are unavailable or inconclusive. Beyond these, AI also leverages the vast data generated in pathology to uncover novel patterns and correlations that may advance patient care.

These efforts fall into three core analytical categories: **segmentation tasks** (*e.g.*, cells and tissue types or lesion boundaries), **diagnosis/prognosis tasks** (*e.g.*, at the WSI- or patient-level), and **discovery tasks** (including cross-modal analyses), supported by auxiliary models that address challenges in their development and deployment.

### Segmentation tasks

**Fig 6:**
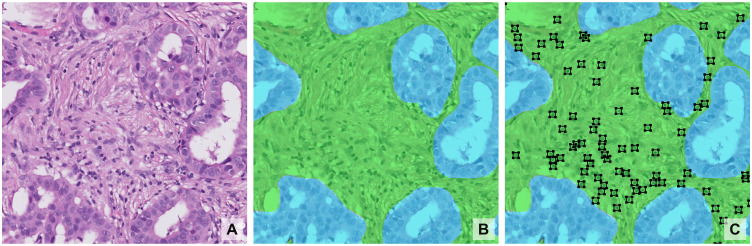
(A) Breast tumour tissue, (B) with semantic segmentation of invasive tumour (light blue) and tumour-associated stroma (green), and (C) instance detection of immune cells (black boxes). Example from the TIGER [25] challenge.

Segmentation in digital pathology involves locating and labelling regions or objects within a WSI. This usually takes the form of **semantic segmentation**, usually used for tissue regions, or **instance segmentation**, usually used for specific types of cells (Fig 6). For these tasks, the ground truth can be precisely established: the pathologist can see and manually annotate a specific region or object on the slide, and the AI model aims to learn to do the same. However, such pixel segmentations are labour-intensive, thus many studies focus on tasks with public datasets, commonly associated to open challenges, such as CAMELYON [24] for breast cancer metastasis detection in lymph nodes, TIGER [25] for tumour and stroma segmentation (Fig 6B), GLAS [26] for colorectal gland segmentation, ICIAR BACH [27] for breast cancer grading or PANDA [28] for prostate cancer grading. Instance segmentation targets smaller, sparser elements, often requiring prolonged search to annotate, also leading to a focus on tasks with publicly available data. Examples include immune cells [25] (*e.g.*, Fig 6C), mitotic figures for breast cancer prognosis [29], and nuclear segmentation (*e.g.*, MoNuSeg [30]). Instance segmentation is also used for immunohistochemically stained slides, where AI can quantify protein expression (*e.g.*, PD-L1 in lung cancer) more precisely than visual estimates [31].

Common models used for the segmentation tasks include U-Net and its variants (nnU-Net [32], pathology-nnU-Net [33]), cell-specific architectures like HoVer-Net [34], HoverNext [35], and StarDist [36], and increasingly, transformer-based approaches (*e.g.*, Swin Transformer [37]), sometimes enhanced by general or pathology-specific foundation models.

**Fig 7:**
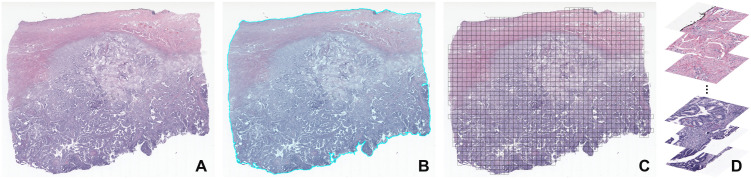
(A) A cervical carcinoma WSI, (B) its tissue mask (light blue), and (C) a visualisation of where (D) tissue tiles (1024 × 1024 pixels) would be taken to feed a model. Example from The Cancer Genome Atlas (TCGA) dataset [16].

Due to the very large size of a typical WSI, slides must first be split into equally sized images, called **patches** or **tiles**, taken at the magnification considered best for the application and with a size optimised for computational resources and model design (Fig 7). The model is then applied to each individual tile, producing a patch segmentation that can be stitched back together to form a full slide segmentation, as a standard procedure implemented by several WSI-processing open-source libraries, such as the TIAToolbox [38], MONAI [39], or PathML [40].

### Diagnosis and prognosis tasks

While segmentation focuses on localising specific morphologies, diagnosis and prognosis tasks predict **slide-** or **patient-level information** without explicitly delineating regions. An example of such a task would be the prediction of liver cell enlargement, *i.e.,* hypertrophy, in drug safety studies, whose presence is more important to detect than its location for assessing drug toxicity. Diagnosis and prognosis tasks can also span multiple WSIs, such as when diagnosing the ISUP (International Society of Urological Pathology) grade of a prostate tumour to determine if systemic treatment is necessary. This requires the analysis of all the tissue from the prostate, which spans multiple slides.

Such an approach can also be used to predict patient prognosis, such as **overall survival** or **treatment response**. In these cases, since no pixel-level annotations are required, ground-truth labels can often be obtained from pathology or patient reports. Such weak labels make patch-wise segmentation-style approaches unreliable, given that only a small portion of pixels may be relevant. Instead, weakly supervised, *e.g.*, multiple instance learning (MIL), is typically used: features are extracted from all patches using a large pre-trained network, aggregated across the WSI or patient, and then classified. Aggregation modules often employ attention or clustering. Popular pipelines include ABMIL [41], CLAM [42], TransMIL [43], and HIPT [44,45], while feature extraction increasingly relies on pathology foundation models such as CTransPath [46], Virchow2 [47], Phikon [48] and UNI [49], which offer strong generalisation for diverse diagnostic and prognostic tasks [50,51].

Building on this, the new frontier for computer-aided diagnosis and prognosis goes beyond simple WSI-only outputs [52]. It involves the development of **visual question answering** tools (*e.g.*, SlideChat [53], PathChat [54]) and the **automated generation of pathology reports** and image captions (*e.g.*, PRISM [55], HistoGPT [56]). The goal of this approach is to emulate the processing and synthesis of visual and contextual data involved in routine pathologic assessment and to create an end-to-end diagnostic system for pathologists to use.

### Discovery tasks

While segmentation and diagnosis/prognosis tasks automate existing pathology workflows, discovery tasks aim to uncover novel links between pathology data and other biomedical modalities. Examples include cross-modal registration between WSIs and radiology images (*e.g.*, prostate cancer [57]) and predicting genetic alterations directly from tissue morphology, first demonstrated in lung cancer [58] and later extended to multiple cancers [59], and preclinical studies [60]. These findings revealed previously unknown genotype–phenotype relationships and inspired the development of pathology-specific foundation models capable of robust performance across diverse tasks. Approaches vary: known-label problems such as mutation prediction often use weakly supervised methods with explainability tools (*e.g.*, Grad-CAM [61]); cross-domain translation employs generative models (*e.g.*, StyleGAN [62]); and foundation models typically adopt transformer architectures trained with self-supervised methods common in natural image analysis (*e.g.*, DINOv2 [63]).

### Supportive tasks

Developing AI models for core analytical tasks in digital pathology often requires substantial data preparation, much of which can be automated with supportive AI models. For example, WSIs contain large non-tissue regions that dilute the diagnostic signal and slow processing. These can be excluded using classical image processing (*e.g.*, Otsu thresholding [64]), though such methods struggle with low tissue–background contrast, such as many IHC-stains and fatty tissue areas, prompting the development of AI-based **tissue–background segmentation** models [65,66].

**Stain normalisation** is another common preprocessing step. Variations in stain appearance, driven by site-specific protocols and preferences, can add noise without reflecting biological differences. Traditional approaches, such as the Macenko normalization [67], adjust stain concentrations but may fail with overlapping absorption spectra, as is common with H&E, leading to AI-based colour normalisation methods that are more robust across staining types [68,69].

Supportive AI models also assist with **ground-truth generation** for segmentation. Semi-automated approaches use pretrained models to produce initial segmentations, which are then corrected by pathologists. Pathologist-in-the-loop systems further refine models iteratively, reducing manual workload. Several open-source (*e.g.*, VGG Image Annotator [70,71], SAM-for-Qupath [72]) and commercial tools (*e.g.*, HALO [73], Discovery [74], and Aiforia Create [75]) employ this strategy.

Similarly, some models can also extract labels directly from patient or lab reports. Finally, supportive AI plays a role in deployment, such as **WSI quality control**. Routine-care slides may contain artefacts that compromise AI performance, and these specialised models can detect and exclude such slides (or even just affected regions within the slide) to maintain reliability in clinical use [76].

### Challenges when developing AI for pathology

When developing AI models for pathology, several challenges are typically encountered. Some of these stem from the size and structure of WSIs, some are inherent to applying AI in a medical context. In Table 1, we summarise the most common ones for both sources. We then provide some information on the typical solutions and their limitations. Addressing these limitations is an active area of research. For example, to mitigate performance degradation caused by tiling WSIs for slide- or patient-level tasks, several MIL approaches have proposed sophisticated tile aggregation methods that help restore some of the context lost. To tackle the inherent data variability of the slides, there is an active effort to choose models that are invariant to such batch effects, rather than relying on additional preprocessing and quality control. One common strategy involves mapping the feature space of a dataset to a lower dimension (*e.g.*, using t-SNE) and examining whether there are clusters that correspond to unintended sources of variability (*e.g.*, centre, preparation method, scanner). Together, these efforts aim to enhance the reliability and usability of AI models developed for pathology, moving these novel technologies a step closer towards translation.

**Table 1:** Typical challenges when developing AI for pathology.

Challenge	Solution(s)	Limitation(s)
WSIs are too large to process as a whole and end-to-end in typical AI pipelines	**Tiling**: splitting the WSI into smaller images that are fed to an end-to-end network one after the other, as shown in Fig 7	Performance degradation due to context loss, slow training, and deployment
	**Streaming** [77,78]: the first layer activations are computed tile by tile until the entire WSI is covered; this is repeated for subsequent layers, so the model considers the entire image before error computing	Computationally expensive, slow to train, and limited to convolutional neural networks
WSI preparation variability [79]	**Preprocessing** and **quality control**	Additional deployment burden
Imperfect pathologist-generated ground truth	**Consensus**: multiple pathologists discuss ground truth options until consensus is reached	Expensive and time consuming
	**Outcomes as labels**: using overall survival or progression-free survival as labels	Label uncertainty (*e.g.*, pseudo-progression, loss to follow-up)
Unbalanced labels	**Class sampling**: target the less frequent class for sampling, sample classes equally	Not possible to get enough data if population incidence is low
	**Loss function adjustment**: weighing the minority class more heavily	Limited if there are insufficient minority class examples
Unbalanced demographics (*e.g.,* gender, age, strain, environment)	**Disaggregated performance evaluation**: evaluate and report performance metrics for each subset	Requires population factor identification, and some information may not be available for all
Non-representative training population	**Prospective evaluation**: test the model on a prospective sample	Expensive and may still give limited representation

## Conclusion

By merging the diagnostic expertise of pathology with the analytical power of computer science, computational pathology stands as a powerful link between medicine and technology, improving disease comprehension and diagnosis. To make sure that innovations resonate with pathological relevance and clinical applicability, continuous interdisciplinary collaboration is essential, as promoted by the European Society of Digital and Integrative Society (ESDIP). To that end, this guide aims to promote a common language between medical and computational experts by outlining the fundamental concepts and processes from tissue processing to digital imaging, from diagnostic reasoning to algorithmic modelling. With this, we hope to provide a starting point for technical experts from various backgrounds to gain an overview of the field and to take the first step towards translating their expertise into the field of pathology.

## Supporting information

S1 Appendix Supporting information. **Table A.** Common artefacts in the histopathology workflow. **Fig A.** Cautery artifact details.
(PDF)
