# Supplementary material for: Decoding (digital) histopathology: The building blocks for computational researchers
Source: PLOS Digit Health. 2026 May 13;5(5):e0001148. doi: 10.1371/journal.pdig.0001148 (PMC13170827; doi:10.1371/journal.pdig.0001148)
Supplement: S1 Appendix — Table A. Common artefacts in the histopathology workflow. Fig A. Cautery artifact details. (PDF) [file pdig.0001148.s001.pdf]

Supporting Information

Step	Artifact	Description
Tissue collection	Cautery artefact[1]	Swelling and homogenization of connective tissue fibres, vacuolisation, and blurring of nuclei due to thermal stress induced by the collection method.
	Crush artefact[2]	Tissue distortion caused by compression, either during tissue handling or instrument use.
Fixation	Tissue lysis[3,4]	Generally due to underfixation, causes altered, suboptimal morphology of the glass slide and can alter the preservation of antigens within tissues for IHC analysis.
Grossing	Cassette-induced clefts	The shape of the tissue may be distorted by the cassette.
	Gel or sponge contaminants[5–7]	Additional material used for orientation (*e.g.* gels, sponges) may be present in the final slide.
Embedding	Malpositioning or misalignment	When the sample is embedded suboptimally, downstream artefacts can follow, including suboptimal microtome cutting, missing tissue pieces, and impaired microscopic examination and thus diagnosis.
Cutting	Tissue folds[8]	When the collection from the water bath is not perfect, the tissue may fold upon itself and hinder staining and observation.
	Venetian blind[9]	Parallel strips of tissue separated by clear spaces, resembling a venetian blind.
	Tissue beyond coverslip[10]	Situation where a tissue sample extends beyond the area covered by a coverslip on a microscope slide. It can lead to issues in microscopy and digital slide scanning, including incomplete scanning of the tissue and potential artifacts. Furthermore, if the slice is not picked up at the centre of the slide, but on the edges, part of the tissue may not be in the scanning area (and thus will not be present in the WSI).
	Irregular thickness[11]	Artifact that can occur during the microtome sectioning process. This issue can negatively impact the quality of histological slides and staining, making it difficult to accurately analyse tissue features.
	Tissue scratches	Artefacts that appear as straight or ragged lines on a tissue section due to flaws, dirt, or vibrations in the cutting edge of the microtome.
	Contaminants[12]	If the water bath is not cleaned up, extraneous material (*e.g.* tissue from previous cases) might be picked up and placed on the glass slide.
Coverslipping	Misaligned coverslip[13]	Coverslip hangs over the edge of the glass slide, creating problems with the mechanical movement of the slide scanner.
	Multiple overlapping coverslips[13]	Multiple glass coverslips might be dispensed (instead of one), resulting in a thicker layer with worse optical properties.
	Bubbles[13]	When the mounting medium does not cover the full surface between the slide and the coverslip. This may result in difficulties both for optical microscopy and for scanning.

Table A. Common artefacts in the histopathology workflow.

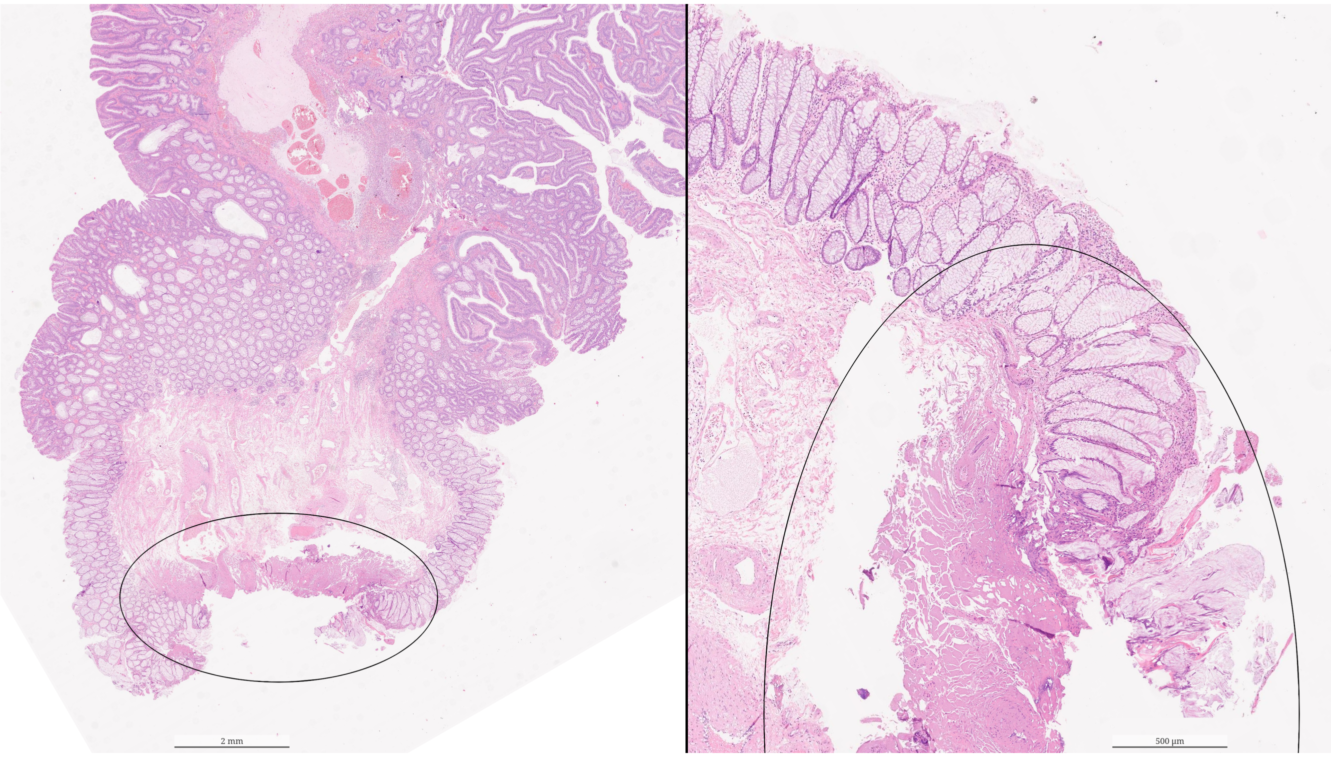

Figure A. Detail of cautery artifact on a colorectal polyp H&E sample. The cauterized area (circled) shows denser amorphous stroma and coagulated glands with loss of detail.

References

1. Lagerveld, B. W., Koot, R. A. & Smits, G. A. Thermal artifacts in bladder tumors following loop endoresection: Electrovaporization v electrocauterization. *J. Endourol.* **18**, 583–586, DOI: 10.1089/end.2004.18.583 (2004).
2. Flamminio, F., Tosi, A. L. & Fellegara, G. Crushing artifacts resulting in small blue cellular clusters that simulate small cell carcinoma. *Int. J. Surg. Pathol.* **19**, 487–491, DOI: 10.1177/1066896911411187 (2011).
3. Goldstein, N. S., Ferkowicz, M., Odish, E., Mani, A. & Hastah, F. Minimum formalin fixation time for consistent estrogen receptor immunohistochemical staining of invasive breast carcinoma. *Am. J. Clin. Pathol.* **120**, 86–92, DOI: 10.1309/qphdrb00qxgmuq9n (2003).
4. Canadian Association of Pathologists-Association canadienne des pathologistes National Standards Committee *et al.* Canadian association of Pathologists-Association canadienne des pathologistes national standards Committee/Immunohistochemistry: best practice recommendations for standardization of immunohistochemistry tests. *Am. J. Clin. Pathol.* **133**, 354–365 (2010).
5. Landas, S. & Bromley, C. Sponge artifact in biopsy specimens. *Arch. pathology & laboratory medicine* **114**, 1285–1287 (1990).
6. Kepes, J. J. & Oswald, O. Tissue artefacts caused by sponge in embedding cassettes. *The Am. J. Surg. Pathol.* **15**, 810–812, DOI: 10.1097/00000478-199108000-00013 (1991).
7. Farrell, D. J., Thompson, P. J. & Morley, A. R. Tissue artefacts caused by sponges. *J. Clin. Pathol.* **45**, 923–924, DOI: 10.1136/jcp.45.10.923 (1992).
8. Palokangas, S., Selinummi, J. & Yli-Harja, O. Segmentation of folds in tissue section images. In *2007 29th Annual International Conference of the IEEE Engineering in Medicine and Biology Society*, 5641–5644, DOI: 10.1109/iembs.2007.4353626 (IEEE, 2007).
9. Joshi, R. "venetian blinds" artifact in dermatohistopathology. *Indian Dermatol. Online J.* **3**, 59, DOI: 10.4103/2229-5178.93497 (2012).
10. Patel, A. U. *et al.* Types and frequency of whole slide imaging scan failures in a clinical high throughput digital pathology scanning laboratory. *J. Pathol. Informatics* **13**, 100112, DOI: 10.1016/j.jpi.2022.100112 (2022).
11. Shah, M. *et al.* Impact of tissue thickness on computational quantification of features in whole slide images for diagnostic pathology. *Endocr. Pathol.* **36**, DOI: 10.1007/s12022-025-09855-2 (2025).
12. Platt, E., Sommer, P., McDonald, L., Bennett, A. & Hunt, J. Tissue floaters and contaminants in the histology laboratory. *Arch. Pathol. Lab. Medicine* **133**, 973–978, DOI: 10.5858/133.6.973 (2009).
13. Ferreira, D., Vale, J., Curado, M., Polónia, A. & Eloy, C. The impact of different coverslipping methods in the quality of the whole slide images used for diagnosis in pathology. *J. Pathol. Informatics* **13**, 100098, DOI: 10.1016/j.jpi.2022.100098 (2022).
